# Comparative Physiology of Menstruation

**Published:** 1884-07

**Authors:** Alfred Wiltshire

**Affiliations:** Lond.


### Art. XIX.—THE COMPARATIVE PHYSIOLOGY OF MENSTRUATION.

BY ALFRED WILTSHIRE, M.D., F.R.C.P., LOND.

[From the London Veterinary Journal.]

Continued from Page 171.

Trousseau (*Clin. Med.*, p. 598), speaking of signs exhibited by the lower animals while breeding, says :—" Need I add that, during the period of rut in most female animals, the congestion of the genitals manifests itself by a flow of blood, and by an increase in the secretion of the glands, which are annexed to those organs?" Both Tarnier and Chantreuil (*Tr. d'Accouch.*), and Cazin, in his memoir on "Varices in Pregnancy and Parturition" (*Arch. de Zool.*, 1880), quote Raimond as stating that, "In the females of animals the vagina is colored red at the epoch of heat; it takes a violet or brownish tinge during pregnancy, and the mucous membrane seems to thicken." Cazeaux says :—" In the rabbit it is tumefaction and almost varicose injection of the vessels of the vulva. To this coloring and tumefaction is added, in the bitch, an odorous secretion, which allures the males and puts them upon the track of the females. Finally, in monkeys a more or less abundant hæmorrhage occurs, which, in the case of the macaques and the cynocephalæ, coincides with so monstrous a swelling of the vulva, that, in certain cases, the surrounding parts are infiltrated, as though inflamed in consequence of the sting of bees."

Raciborski (*Traité de la Menstruation*, p. 43) remarks on the resemblance between the rut and menstruation in the matter of periodicity, and states that sows have symptoms every fortnight to eighteen days, heifers every twenty-one days, sheep every fortnight. Quoting from a friend having choice cattle, he says that the higher breeds require the male more often than inferior breeds ; on which he exclaims "*En violà un singulier privilége de l'aristocratic dans la race bovine!*"

Generally, in most animals, there is a swelling of the external genitals, and a discharge which is often sanguinent, especially in the heifer, bitch, sow, and rabbit, while in monkeys, particularly in the great species, it often takes the proportion of a hæmorrhage.

Raciborski regards woman and the quadrumana probably as the only females who have complete exfoliation of the mucous membrane of the uterus after each conception, and considers that the commencement of this important process is repeated at each ovulation. He says, also (p. 18), that he has seen in the Jardin des Plantes, at Paris, monkeys who were abundantly regular every month for five days.

Mr. Darwin incidentally refers to the existence of the periodical function in the females of the lower animals thus (*Animals and Plants under Domestication*, vol. i., pp. 33, 34) : "Several years ago I saw, confined in the Zoological Gardens of London, a female hybrid, from an English dog and jackal, which even in this, the first generation, was so sterile that, as I was assured by her keeper, she did not fully exhibit her proper periods ;" and again (*Ibid.*, vol. ii., p. 132) : "Elephants . . . both male and female, have their proper periodical seasons."

Fleming (*Veterinary Obstetrics*, p. 55) says : "The rutting, heat, œstrum, or venereal œstrum of animals is analogous to menstruation in woman, and marks the period of maturation in the ovarian ova or ovum, according to species. This condition is intermittent or periodic, not continuous ; and is characterized by a peculiar systemic excitement, that usually continues for a somewhat definite period in the two sexes. In the male and female, but especially in the latter, the generative organs at this period become more or less turgid and sensitive, and the uro-genital secretions are increased. In the female there is a determination of blood to the ovaries, and changes take place in these which have already been described. The excitation of the generative apparatus reacts on the whole system, and produces a kind of fever or irritability in the animal; its sensibility is increased; the appetite is more or less in abeyance, or capricious, and usually there is thirst. . . . Attempts at micturition in the female are frequent, but only a small quantity of urine is passed; the mucous membrane of the vagina is injected; and, with solipeds, there are oft-repeated movements of the clitoris and vulva, and an opaque white secretion, or even emissions of blood are ejected spasmodically, *per vulvam*." In a foot-note Fleming says: "Kaiser in the *Magazin für die Gesammte Thierheilkunde* for 1859, mentions a mare, twenty-four years of age, which every three weeks had a sanguineous emission from the vulva; this discharge ceased toward the middle of pregnancy, but returned after parturition. I have frequently witnessed the periodic discharge from mares either streaked with blood, or blood-tinted;" and then, resuming the text, "in other animals, this ejection sometimes consists of a viscid red tinted or sanguinolent fluid. It has a special and powerful odor, which attracts the males. . . . The uterine mucous membrane is also very congested, and there is poured out on its surface a fluid containing epithelial *débris*, mucous, corpuscles, and blood-globules.

"The existence in the lower animals of what is analogous to the menstrual discharge in the human female has frequently been denied, but without any reason or proof. A discharge of blood from the sexual organs of woman announces the advent of puberty; and its coincidence with the maturity and escape of the ovarian ovule, as well as its periodical appearance until the termination of fertility, establishes between this phenomenon and the heat or 'rut' (œstrum) of animals a very close analogy. Kahleis, Fuschs, Spinola, Fumann, and others, have observed this in the cow, and have also noted that the discharge occurs regularly at intervals of nineteen or twenty days when the animal is not giving milk or in calf. The hæmorrhagic flow appears two or three days after the commencement of 'rutting,' and when this is most intense. The amount of blood does not exceed one or two ounces, and the coagulated clot remains in the vagina until it is expelled with the urine. There can be no doubt as to its source. If at the moment when traces of it are perceived externally, the cow is killed, and the inner surface of the uterus examined, blood will be seen exuding from the cotyledons. And this phenomenon has been proved to extend beyond the bovine species, for it has been witnessed in the mare, bitch, cat, rabbit, etc.; and in the red-colored mucous of the vagina and uterus multitudes of blood-corpuscles have been found.

"With regard to the season at which this 'heat' takes place, it has been observed that it is usually in the spring-time, when food becomes plentiful, especially with herbivorous animals. The carnivora are in heat during winter. The mare is usually in heat from April to June, or later. With the cow, whose calf is sold at from one to two months old, with a view to utilizing the milk, the season, of course, is varied—as care is taken to induce conception again as soon as the lacteal secretion begins to diminish; but it has been observed that midsummer is more particularly the rutting period. And the 'heat' in sheep, though naturally present in September, is usually only shown during summer, because the ewes are kept apart from the ram at the natural time, in order that the lambs may be born at a favorable season—the spring. . . . . The bitch is in heat from December to February, or in the autumn or spring-time. The cat is in this state in January and February, and also in the spring and autumn; sometimes the heat appears three or four times in a year, and the animal may produce young as frequently, though, in the wild state, it seldom does so more than twice a year. The pig manifests rutting in October or November—at least, that is the period when it is usually put to the male; and it may be put a second time towards the end of spring, in order to have two litters within the twelve months. The frequency and duration of the period of 'rutting' or 'heat' depend upon age, species, and other circumstances; but it may be said to persist, in the domesticated animals, from one to fifteen days at the most. The shortest period is witnessed in the cow and sheep, and the longest in the bitch. It is sometimes only present from twelve to twenty-four hours in some non-fecundated animals. With impregnation, however, it ordinarily ceases until after parturition ; and if impregnation does not occur, it gradually disappears until the next period, which is somewhat variable. Its reappearance in the cow has been noted every month or three weeks, and sometimes at closer intervals ; and in the sheep and pig it lasts for one or two days, and again appears from the fifteenth to the thirtieth day, but usually every month. When removed from artificial conditions, it is stated that the ovine species is in rut in September, that this persists only for a day, but reappears every fourteen days until the end of December. From the spring until the end of summer, it may be said the mare manifests a desire for the horse every three or four weeks, and the objective phenomena which announce it continue from two to four days. In the bitch they last for nine or ten days, and only appear in the spring and autumn. . . . . Domesticity, in assuring animals food and shelter, and removing them from risks and alterations of an erratic life, multiplies the returns of this condition. Fowls, pigeons, etc., lay despite the rigors of winter, and the domesticated mammals are in heat at short intervals. . . . . Some animals, as the mare and pig, manifest a desire for the male, and even copulate (shortly after delivery) ; and it is no less a fact that rutting and impregnation may, and do occur, soon after parturition. The cow, ass, and sheep, and, it is believed, the mare will copulate with greater certainty of success the ninth day after parturition than at any other time."

Saint-Cyr (*Traité d'Obstétrique Vétérinaire*, page 41) says that the females of domesticated animals show signs of excitement during the period of heat—not only by general symptoms, but by swelling of the vulva, and an odorous glairy or sanguinolent discharge ; and that, in the cow particularly, and frequently in the mare, this discharge is abundantly sanguineous. He says that this sanguineous excretion, which has been studied with care by Numann, Fuschs, Spinola, and others, is a normal phenomenon, and analogous to the catamenial flux of woman.

Denman (*Midwifery*, page 88) says : "It has also been observed that all animals, at the time of their being salacious, or in a state fit for the propagation of the species, have a discharge equivalent to menstruation, which is generally mucous; but in some instances, in very hot seasons and climates, becomes in many of them sanguineous, as I have often observed."

The celebrated Dr. Mead wrote a treatise on the "Influence of the Sun and Moon on Human Bodies," wherein, according to the prevailing notions of the time, he labored to show that those great luminaries exerted direct influence upon animals; and, speaking of this power over periodical hæmorrhages, he says: "And this action of the moon extends even to those quadrupeds that are menstruated, for it has been observed that they generally have those evacuations about the new moon—in particular, mares and monkeys—and that so constantly that, according to the testimony of Horus Apollo, the Egyptian painted the cynocephalus to represent the moon, upon account of a certain sympathy, whereby the female of this animal has evacuations of blood from the uterus at the new and full moon; and they kept monkeys in their temples in order to point out the times of the conjunction of the sun and moon. . . . Wherefore, the moon's influence is apparent in all animals, provided irregularities in their way of living do not prevent it."

My own personal inquiries into the character of the phenomena observable in females of the lower animals at their times of sexual excitement, show that a more or less marked sanguineous flux occurs in the following: Monkeys, mares, cows, sows, and bitches. In the higher monkeys, when they do not suffer through captivity, the flow is almost—sometimes quite—as copious as in women. In some varieties the genitalia becomes swollen and brilliantly colored, so that tomato-like vermillion-tinted masses render their condition conspicuous. The simiæ also commonly observe monthly regularities in their periods. In mares the ' pouting vent," with its incessant spasmodic opening and closing, and its rosy-colored, highly odorous, sanguino-mucous weeping, proclaims the period of heat. In the cow the period recurs every three weeks, and is of brief duration; but towards its conclusion it is marked by a distinct flow of blood. Mr. Simpson, of Wray Park, whose accurate observations upon his splendid herd of Jersey cattle during fifteen years may be implicitly relied upon, informs me that the duration of the rut in old cows is briefer than in heifers, but is short in both ; and his herdsmen have often to be nimble in getting the service of the bull. When the blood appears the heat usually subsides, and intercourse is refused. Cows have been known to have excessive loss, resembling the menorrhagia of women. Mr. West confirms these observations from a very wide experience as a veterinary surgeon.

It may here be appropriate to mention that evidence is not wanting to show that the menstrual act in milch cows is apt to derange the creatures' milk temporarily, in some instances rendering it unfit for consumption by suckling infants. It is maintained by certain observers that the milk of menstruating women also deteriorates during the catamenia, when the latter occur during lactation ; and I have met with many corroborative examples, though in other cases no particular harm appears to have ensued. The probability of derangement arising from this cause receives some confirmation from the changes which are known to take place in human milk at the catamenial periods. Dr. Barnes states that microscopical examination of milk yielded by menstruating women shows a considerable production of colostrum corpuscles at these epochs. Such milk seems to resemble the first secreted after delivery (colustrum), and in cows this first milk, or "beestings," is known to possess very peculiar properties. It is, for instance, in them so highly albuminous as to coagulate firmly on the application of heat; the resulting *magma* resembling pretty closely custard-pudding; indeed, in country places I have known "beestings" used for the purpose of making such puddings; and, as in women, this first milk is believed to exert rather an aperient effect upon the offspring. Its color is much yellower, and its aspect is very different from that of ordinary milk. My inquiries lead me to believe that, besides the alterations recognizable microscopically, there are other, perhaps more important, changes in the composition of menstrual milk, affecting materially not only its corpuscles, fat, and casein, but its chemical constituents, salts, sugar, etc. Probably it is to impairment of the quality of the milk by subtle changes of the kind indicated that the deleterious results are attributable.

Acute emotions of a distressing character, particularly violent anger and jealousy, have been known to alter a mother's milk so completely as to excite symptoms of sudden poisoning, and even convulsions, in the infant.* The secretion must therefore obviously be highly susceptible of rapid modification in its structure and composition.

Probably, in a herd of dairy cattle, the admixture of the milk of one or two rutting cows with that of the rest of the herd is of but little consequence ; and fortunately, as already stated, the duration of the rut in ruminants is usually brief. Besides, if I am rightly informed, milch cows, like women, do not always manifest symptoms of œstruation during, at any rate, the earlier months of lactation, though they commonly do, and the heat may supervene at any time. But when, as of late has become a growing custom amongst well-to-do people, hand-fed infants have milk supplied exclusively by one cow, periodical disturbance may be manifested in the infant, corresponding with observed periods of heat in the animal.

In sows the vulvar swelling is considerable, and a sanguineous exudation may appear towards the termination of the period of heat, particularly in well-fed animals. In bitches the flux is often abundant and prolonged. Mr. Bartlett informs me that the surest method for procuring impregnation in the bitch is to permit access to the male only when the flow of blood is well established—a statement which is corroborated by information for which I am indebted to Mr. Bird, J. P., who has bred many bloodhounds. This has some bearing upon most questions relating to impregnation, conception, and ovulation. In the well-fed carnivora the heat appears to be sometimes prolonged, when not appeased by sexual gratification.

Besides the fore-mentioned creatures, personally observed, information respecting other female animals has been given me by reliable observers. Thus, the periods of heat have been recognized in the hippopotamus in the Zoological Gardens. These recur monthly, and the creature's period of gestation is

---

* I lately saw a marked instance of this in consultation with Dr. Milson. Instances of this have lately been recorded by Dr. Napier (*Lancet*, August 19th, 1882), and by Mr. Walford (*Lancet*, September 2d, 1882) ; and my own inquiries have yielded support to this view. Two infants, supplied exclusively from separate cows, showed periodical symptoms of bowel-disorder, so that, in each instance, those in charge of them requested that the children might be supplied from the mixed milk of the dairy instead of from selected cows.

only seven months, which strikes me as remarkably short for so huge an animal; it has been accurately noted, however, on three occasions. The smallest tapir, which has recently bred in the Gardens, is believed to gestate for double this period. Such apparent anomalies show the importance of inquiry into specific or generic peculiarities. In the equine species, which is thought by some to have ancestral affinities with the tapir, the mare goes with young for a long period, her time of gestation being eleven months, while that of the cow is only nine months, as in woman. There is an old saying, known in country places, that "A hare and a mare go a twelve months"—that is, the hare goes one month, while the mare goes eleven.

I am told that she-asses come to heat regularly like mares, but that the periods recur only about every five weeks. They also exhibit swelling and weeping of the vulva. Sheep present similar phenomena, but they are not strongly marked, and recur only seasonally—*i. e.*, in warm months—for a few times, and are of brief duration. Cats are known to have recurring periods of heat, but how often I am not reliably informed. Doe rabbits are well known to manifest periodic heat-symptoms, and their vulva orifice, vagina, etc., show deep purple turgescence.

It is evident that animals susceptible of domestication become much more prolific under the agency of a constant supply of food, shelter, and such-like advantages, and that, in lieu of the merely seasonal manifestations of sexual aptitude displayed by them in a state of nature, they acquire a much more sustained, and frequently renewed, capacity for reproduction, and with it comes the exhibition of aptitude in the characteristic manner peculiar to each creature. As Mr. Darwin says, "Domestication for the lower females, and civilization for the human female, have respectively so operated upon their reproductive systems as to enhance their powers and repeat, in the intensification, the frequent display of catamenial phenomena.

The foregoing evidence appears sufficient to establish the fact that the lower females present phenomena analogous to those of menstruation in the human female, and even to prove their identity. The facts also seem to justify the chief inference I have ventured to draw from them, namely, that the function is insubordinate to the law of evolution, and is related mainly to the degree of evolution to which the sexual system has attained, such evolution being always correlated with other evidences of organic development. They enlighten us respecting the correlations of the reproductive functions in the mammalian world; they show us the singular analogies that exist between the seasonal rutting and delivery of the lower females, and the still observable tendency to commence menstruation, and to bring forth children, in the warmer months, of the human female, that relic of a primordial seasonal aptitude for procreation, the impress of which still remains, and, to some extent, governs the breeding times of humanity. They show us that the rare menstruation at long intervals—six, four, or three months—of girls at puberty, is analogous with the primitive seasonal heat of the lower creatures, and is a repetition of a reversion to an ancestral condition, often imitated again at the decline of sexual life. These and allied facts will find their application, and be constantly appealed to in illustration of the physiology and pathology of the function in womankind; and I trust it will become clear to you that the information acquired through the study of the comparative physiology of menstruation is neither superfluous nor useless, either in the scientific discussion or in the practical application of our knowledge of the subject.

